# Co-Occurrence of *Francisella*, Spotted Fever Group *Rickettsia*, and *Midichloria* in Avian-Associated *Hyalomma rufipes*

**DOI:** 10.3390/microorganisms10071393

**Published:** 2022-07-11

**Authors:** Tove Hoffman, Andreas Sjödin, Caroline Öhrman, Linda Karlsson, Ryelan Francis McDonough, Jason W. Sahl, Dawn Birdsell, David M. Wagner, Laura G. Carra, Peter Wilhelmsson, John H.-O. Pettersson, Christos Barboutis, Jordi Figuerola, Alejandro Onrubia, Yosef Kiat, Dario Piacentini, Thomas G. T. Jaenson, Per-Eric Lindgren, Sara Moutailler, Thord Fransson, Mats Forsman, Kenneth Nilsson, Åke Lundkvist, Björn Olsen

**Affiliations:** 1 Department of Medical Biochemistry and Microbiology, Zoonosis Science Centre, Uppsala University, Husargatan 3, 751 23 Uppsala, Sweden; lauracarra91@gmail.com (L.G.C.); john.pettersson@imbim.uu.se (J.H.-O.P.); ake.lundkvist@imbim.uu.se (Å.L.); 2 CBRN Defence and Security, Swedish Defence Research Agency, Cementvägen 20, 906 21 Umeå, Sweden; andreas.sjodin@foi.se (A.S.); caroline.ohrman@foi.se (C.Ö.); linda.karlsson@foi.se (L.K.); mats.forsman@foi.se (M.F.); 3 Pathogen and Microbiome Institute, Northern Arizona University, Flagstaff, AZ 86011, USA; ryelan.mcdonough@nau.edu (R.F.M.); jason.sahl@nau.edu (J.W.S.); dawn.birdsell@nau.edu (D.B.); dave.wagner@nau.edu (D.M.W.); 4 Department of Biomedical and Clinical Sciences, Division of Inflammation and Infection, Linköping University, 581 85 Linköping, Sweden; peter.wilhelmsson@liu.se (P.W.); per-eric.lindgren@liu.se (P.-E.L.); 5 Department of Clinical Microbiology, Region Jönköping County, 551 85 Jönköping, Sweden; 6 Sydney Institute for Infectious Diseases, School of Life and Environmental Sciences and School of Medical Sciences, The University of Sydney, Sydney, NSW 2006, Australia; 7 Antikythira Bird Observatory, Hellenic Ornithological Society/BirdLife Greece, 10437 Athens, Greece; cbarboutis@ornithologiki.gr; 8 Estación Biológica de Doñana, CSIC, Avda. Américo Vespucio 26, 41092 Sevilla, Spain; jordi@ebd.csic.es; 9 CIBER Epidemiología y Salud Pública (CIBERESP), 28029 Madrid, Spain; 10 Migres Foundation, P.O. Box 152, 11380 Tarifa, Spain; aonrubia@fundacionmigres.org; 11 Israeli Bird Ringing Center (IBRC), Israel Ornithological Center (IOC), Society for the Protection of Nature in Israel (SPNI), Tel-Aviv 6618602, Israel; yosefkiat@gmail.com; 12 Independent Researcher, Via Cesare Lippi 35, 40026 Imola, BO, Italy; dariocarmen@alice.it; 13 Department of Organismal Biology, Evolutionary Biology Centre, Uppsala University, Norbyvägen 18d, 752 36 Uppsala, Sweden; thomas.jaenson@ebc.uu.se; 14 ANSES, INRAE, École Nationale Vétérinaire d’Alfort, UMR BIPAR, Laboratoire de Santé Animale, F-94700 Maisons-Alfort, France; sara.moutailler@anses.fr; 15 Department of Environmental Research and Monitoring, Swedish Museum of Natural History, 104 05 Stockholm, Sweden; thord.fransson@nrm.se; 16 Department of Medical Sciences, Section of Clinical Microbiology, Uppsala University, 751 85 Uppsala, Sweden; kenneth.l.nilsson@medsci.uu.se; 17 Department of Medical Sciences, Zoonosis Science Centre, Uppsala University, 751 85 Uppsala, Sweden; bjorn.olsen@medsci.uu.se

**Keywords:** African-Western Palaearctic region, migratory birds, ticks, *Hyalomma rufipes*, *Francisella*, *Francisella*-like endosymbionts, spotted fever group *Rickettsia*, *Rickettsia aeschlimannii*, *Midichloria*, PCR, metagenomics

## Abstract

The migratory behavior of wild birds contributes to the geographical spread of ticks and their microorganisms. In this study, we aimed to investigate the dispersal and co-occurrence of *Francisella* and spotted fever group *Rickettsia* (SFGR) in ticks infesting birds migrating northward in the African-Western Palaearctic region (AWPR). Birds were trapped with mist nests across the Mediterranean basin during the 2014 and 2015 spring migration. In total, 575 ticks were collected from 244 birds. We screened the ticks for the species *Francisella tularensis*, the genus *Francisella*, and SFGR by microfluidic real-time PCR. Confirmatory analyses and metagenomic sequencing were performed on tick samples that putatively tested positive for *F. tularensis* during initial screenings. *Hyalomma rufipes* was the most common tick species and had a high prevalence of *Francisella*, including co-occurrence of *Francisella* and SFGR. Metagenomic analysis of total DNA extracted from two *H. rufipes* confirmed the presence of *Francisella*, *Rickettsia*, and *Midichloria*. Average nucleotide identity and phylogenetic inference indicated the highest identity of the metagenome-assembled genomes to a *Francisella*-like endosymbiont (FLE), *Rickettsia aeschlimannii*, and *Midichloria mitochondrii*. The results of this study suggest that (i) FLE- and SFGR-containing ticks are dispersed by northbound migratory birds in the AWPR, (ii) *H. rufipes* likely is not involved in transmission of *F. tularensis* in the AWPR, and (iii) a dual endosymbiosis of FLEs and *Midichloria* may support some of the nutritional requirements of *H. rufipes.*

## 1. Introduction

Ticks (Acari: Ixodida) transmit pathogens of both human and veterinary importance, such as bacteria in the genera *Anaplasma, Borrelia*, *Coxiella*, *Francisella*, and *Rickettsia.* They also can be co-infected with different pathogens that potentially can cause co-infections in hosts [1,2]. Additionally, ticks harbor endosymbionts living symbiotically within them. Bacterial endosymbionts of ticks are mostly from the genera *Coxiella*, *Francisella*, and *Rickettsia*; they are closely related to pathogens and may be necessary for the survival of the host [3]. Ticks are strictly hematophagous, meaning their diet consists solely of vertebrate blood, which is nutritionally unbalanced since it contains a high level of proteins but few vitamins [4]. Endosymbiotic bacteria present within tick cells are believed to support the dietary requirements of ticks by providing nutrients that are absent in vertebrate blood [5,6].

The genus *Francisella* includes both pathogenic and non-pathogenic species, including endosymbionts [7]. *Francisella* has previously been divided into two major genetic clades [8], but recently four major clades have been recognized (Clade 1–4) [7]. *Francisella tularensis* and *Francisella*-like endosymbionts (FLEs) are assigned to Clade 1 [7]. *F. tularensis* is primarily present in the Northern hemisphere and has a broad host range, including mammals, birds, and arthropods [9]. Furthermore, *F. tularensis*, the causative agent of tularemia, is regarded as a potential agent of biological warfare [10]. Infection in humans is acquired via direct contact with infected animals, ingestion of contaminated food or water, inhalation of contaminated particles, or bites of blood-feeding arthropods (i.e., ticks, tabanids, and mosquitoes) [9]. Multiple tick species from the genera *Amblyomma, Dermacentor, Ixodes*, and *Haemaphysalis* are vectors of *F. tularensis* [9]. The genomes of FLEs include pseudogenes and inactivated versions of virulence genes of *F. tularensis*, suggesting they arose from a pathogenic ancestor [5,11]. FLEs replicate intracellularly and can infect the ovaries of female ticks, enabling transovarial transmission (i.e., from the female tick to her offspring) and ensuring the continuation of the symbiotic relationships [12,13]. FLEs are known to have a broad geographical distribution [14,15,16,17,18,19,20,21,22], and are widely distributed across tick taxa, including both soft (Argasidae) and hard (Ixodidae) tick species, such as *Ornithodoros moubata*, *Amblyomma maculatum*, *Dermacentor andersoni, Dermacentor reticulatus, Dermacentor variabilis*, and *Hyalomma marginatum* [5,15,21,22,23,24,25]. Little is known about FLEs due to culturing difficulties and a limited number of assembled and characterized genomes.

The bacterium *Midichloria mitochondrii* was first described as an endosymbiont of *Ixodes ricinus* ticks known to reside primarily in the ovarian primordia or ovaries, enter the mitochondria, and be transmitted by transovarial transmission to the offspring [26,27,28]. The prevalence of *M. mitochondrii* in female *I. ricinus* ticks has been reported to be 100% [27]. The bacterium also has been detected in ticks of the genera *Hyalomma*, *Rhipicephalus*, *Amblyomma*, and *Haemaphysalis* [29]. DNA of *M. mitochondrii* and of bacteria related to *M. mitochondrii* has been detected in the salivary glands of *I. ricinus* ticks [30] and in blood samples from canines [31], respectively, and antibodies against *Midichloria* have been detected in blood samples collected from canines and humans bitten by ticks [31,32], indicating potential horizontal transmission of the bacterium.

The genus *Rickettsia* has been divided into four groups [33]. Most tick-borne *Rickettsia* belong to the spotted fever group (SFG), which includes members that are considered to be emerging human pathogens in Europe (e.g., *Rickettsia conorii*, *Rickettsia massiliae*, and *Rickettsia aeschlimannii*) [34,35]. *Rickettsia* species in the SFG are transmitted to humans by multiple tick genera, including *Rhipicephalus, Ixodes*, and *Hyalomma* [34]. Wild birds are frequently parasitized by *Ixodes* and *Hyalomma* ticks, and the migratory behavior of the avian hosts aids in the geographical spread of ticks and their associated microorganisms [36,37,38]. Because they are intracellular tick-borne bacteria, *Francisella*, *Midichloria*, and *Rickettsia* are difficult to culture, and culture-independent generation of genome sequences is of importance for increasing the knowledge and understanding of these bacteria. In this study, we aimed to investigate the dispersal and co-occurrence of *Francisella* and SFG *Rickettsia* (SFGR) species in ticks infesting northbound migrating birds in the African-Western Palaearctic region (AWPR), using microfluidic real-time (q) PCR and metagenomics.

## 2. Materials and Methods

### 2.1. Trapping of Birds and Collection of Ticks

Birds were trapped using mist nets at bird observatories in Spain (several sites in the provinces of Huelva and Sevilla and the Canary Islands: 37°30′ N, 5°30′ W; 37°33′ N, 6°55′ W; 28°9′ N, 15°25′ W), Italy (Capri: 40°33′ N, 14°15′ E), Greece (Crete and Antikythira; 35°51′ N, 23°18′ E), and Israel (Jerusalem and its vicinity: 31°47′ N, 35°13′ E) during their northbound 2014 and 2015 spring migration. Birds were visually inspected for ticks by blowing apart the feathers. Collected ticks were stored in RNA*later*^TM^ (Invitrogen, ThermoFisher Scientific, Waltham, MA, USA) at −20 °C or at refrigerator temperature during the bird ringing season, a period when birds are trapped, measured, weighed, and ringed (i.e., banded) by ringers/ornithologists at bird observatories. After the ringing season, ticks were stored at −80 °C. Only northward migrating birds were included in the study. See Hoffman et al. 2021 [39] for additional details.

### 2.2. DNA Extraction

In brief, absolute ethanol (Sigma-Aldrich, Merck, Darmstadt, Germany) and sterile H_2_O were used for surface sterilization of the ticks before homogenization. Mechanical homogenization was performed using a stainless-steel bead (Qiagen, Hilden, Germany), TRIzol^TM^ (Invitrogen, ThermoFisher Scientific, Waltham, MA, USA), and a TissueLyser II (Qiagen). After homogenization, additional TRIzol^TM^ was added to the homogenate, followed by centrifugation and collection of the supernatant. RNA was isolated and removed using a phase separation technique, in which chloroform (Sigma-Aldrich) was added to the supernatant. Thereafter, DNA was extracted from the organic phase using a back-extraction buffer, inversion, and centrifugation. DNA present in the upper phase was purified using the Nucleospin gDNA Clean up kit (Macherey-Nagel, Bethlehem, PA, USA). The DNA was eluted in DE buffer and stored at −20 °C. For additional details, see Hoffman et al. 2021 [39].

### 2.3. Molecular Screening and Confirmation Analyses

#### 2.3.1. Francisella

Tick extracts were screened for the presence of DNA from the genus *Francisella* and the species *F. tularensis* specifically by microfluidic qPCR (BioMark^TM^ Dynamic Arrays, Fluidigm, CA, USA) and with FopA (genus-specific) and Tul4 (species-specific) primers and probes (Table 1), at the Animal Health Laboratory (Paris, France) according to Michelet et al. [40]. Samples with a cycle threshold (Ct) value higher than 30 were considered negative [40]. To confirm the initial putative findings of *F. tularensis* (Tul4+ samples), subsequent qPCRs were performed using the *Francisella* qPCR assays in Table 1 [40,41,42,43]. In brief, the DNA was pre-amplified (due to the limited amount of DNA) using the RepliG midi kit (Qiagen, Hilden, Germany), according to the manufacturer’s instructions. The PCRs (25 µL) consisted of 2X PerfeCTa qPCR ToughMix (VWR, Radnor, PA, USA), 0.5 µM (final concentration) of each primer, 0.1 µM (final concentration) of each probe, and 1 µL template. The temperature profile was as follows: 95 °C for 10 min, followed by a two-step cycle of 15 s at 95 °C and 60 s at 60 °C. Positive and negative controls were included. The tick DNA samples were tested for PCR inhibitor with an additional qPCR using seal herpesvirus type 1 [44].

#### 2.3.2. Spotted Fever Group *Rickettsia*

Ticks also were screened for SFGR DNA by microfluidic qPCR, using primers and probes targeting the *gltA* gene, according to Michelet et al. [40]. Samples with a Ct-value higher than 30 were considered negative [40]. Confirmation analyses were executed on a small set of ticks (*n* = 38) with Ct-values*_gltA_* ranging from 5.6 to 29.6, using primers targeting the 17 kDa gene by Carl et al. [45] (Table 1). This was done because the confirmation PCR did not include a pre-amplification step, as was the case with the microfluidic qPCR, making it a less sensitive method. In brief, the reaction comprised of 1X Phusion Green HF buffer (ThermoFisher Scientific, Waltham, MA, USA), 200 µM dNTP (Invitrogen, Thermo Fisher Scientific), 0.25 µM (final concentration) of each primer (Invitrogen), 0.02 U/µL Phusion HotStart II DNA polymerase (ThermoFisher Scientific), 8.4 µL sterile H_2_O, and 5 µL template. The reaction profile was as follows: 98 °C for 30 s followed by 35 cycles of 30 s at 98 °C, 30 s at 62 °C, and 30 s at 72 °C, and a final extension at 72 °C for 10 min.

### 2.4. Tick Taxon

Tick taxa and *Hyalomma* species were determined by PCR, using primers by Beati and Keirans [43] (Table 1). See Hoffman et al. 2021 [39] for further details. Morphological determination was not performed since identification of species level of immature ticks belonging to the tick complex *H. marginatum*, including the species *H. rufipes* and *H. marginatum*, is difficult and not recommended [46]. Furthermore, life stage determination was not performed for the infesting ticks. However, the majority of the avian-associated ticks were likely immatures [36,47].

### 2.5. Characterization

#### 2.5.1. Sanger Sequencing

12S rDNA and 17 kDa amplicons were treated with illustra ExoProStar^TM^ 1-step kit (Cytiva, Marlborough, MA, USA), according to the instructions by the manufacturer, prior Sanger sequencing at Macrogen (Amsterdam, the Netherlands).

#### 2.5.2. Spotted Fever Group *Rickettsia*

The CLC Main Workbench 7 by Qiagen (Aarhus, Denmark) was used for assembling partial 17 kDa sequences, which were compared to sequences deposited in the GenBank database [48] using the nucleotide Basic Local Alignment Search Tool (BLASTN) (v.2.10.0) [49].

#### 2.5.3. Metagenomic Sequencing

Two DNA samples putatively positive for *F. tularensis* by microfluidic qPCR were whole genome amplified (RepliG midi kit, Qiagen, Hilden, Germany) before being subjected to further characterization using two sequencing technologies. Non-enriched samples were prepared using TruSeq PCR-free library kits (Illumina, San Diego, CA, USA) and sequenced as 2 × 150 base pairs (bp) in one lane on an S4 flow cell in a NovaSeq 6000 sequencing instrument (Illumina) at the SNP&SEQ platform at NGI Uppsala (Sweden). Enriched samples were prepared using Nextera library kits (Illumina) and sequenced as 2 × 150 bp on a 300-cycle sequencing flow cell using a NextSeq instrument (Illumina) at NAU (Northern Arizona University). Non-enriched samples were also prepared using LSK-109 library kits (Oxford Nanopore Technologies, Oxford, UK) and sequenced in MinION R9.4.1 flow cells using a MinION sequencing device (Oxford Nanopore Technologies).

#### 2.5.4. Enrichment

Due to few metagenomic reads mapping to *Francisella*, RNA baiting was performed at NAU to enrich *Francisella* DNA present in the tick extracts. Briefly, pre-amplified tick DNA extracts were uniquely indexed in ~300 bp sequencing libraries and exposed to RNA hybridization baits (probes) of 120 bp (Agilent Technologies Inc., Santa Clara, CA, USA). The RNA baits were designed against a *Francisella* pan-genome defined by 498 *Francisella* genomes examined in [7]. Sequences <120 bp were removed, and regions with a homology of ≥80% with non-*Francisella* bacteria and ribosomal RNA genes were excluded, yielding 188,430 unique probe signatures, which included 2X tiling to ensure 50% sequence overlap to optimize capture. To further refine optimal capture, manufacturing of replicate copies of ~20,000 probes comprised of high (≥50%) or low (≤22%) GC content was performed. Bait capture was executed twice for increased purification of *Francisella* sequences from background tick DNA.

#### 2.5.5. Taxonomic Classification of Sequence Reads

Sequenced tick samples were characterized with a custom-made database containing bacteria, eukaryotes, and viruses, using Kraken 2 [50]. The database was created using FlexTaxD [51] with bacteria based on the taxonomy from the Genome Taxonomy Database (GTDB) together with eukaryotes and viruses from the National Center for Biotechnology Information (NCBI). The post-processing tool StringMeUp (v.0.1.4) (https://github.com/danisven/StringMeUp, accessed on 15 May 2021) was used for adjusting the results for different confidence scores.

#### 2.5.6. Genome Assembly

##### Enriched Samples

Illumina reads from enriched tick samples were analyzed using a pipeline controlled by Snakemake (v.6.2.1) [52]. Initially, the data were pre-processed by using BBMap (v.38.90) (https://sourceforge.net/projects/bbmap/, accessed on 15 May 2021) to map reads to the collection of 498 *Francisella* genomes [7] that were used in the design of hybridization baits and only keeping mapped reads followed by digital normalization step using bbnorm in BBMap with the settings k = 31 and kmer coverage 100. The remaining reads were de novo assembled using SPAdes (v.3.15.3) [53]. Post-processing of assemblies was performed by removing contigs <500 bp and keeping contigs matching BLAST results containing the ‘*rancisella’ string (to include all different genera inside the family) through BLAST-based filtering. The following blastn settings were used: culling_limit = 5, evalue = 1e-25. Finally, two rounds of Pilon (v.1.24) [54] polishing finalized the assembled sequences before calculating summary statistics using assembly-stats (v.1.0.1) (https://github.com/sanger-pathogens/assembly-stats, accessed on 15 May 2021). The quality of metagenome-assembled genomes (MAGs) was evaluated using checkM (v.1.1.3) [55] and BUSCO (v.5.1.3) [56].

##### Non-Enriched Samples

Illumina reads from non-enriched tick samples were analyzed using the same workflow as enriched samples but with the modification of keeping contigs that matched ‘Rickettsia’ and ‘Midichloria’. Nanopore reads were assembled using Flye (v.2.8.3) [57] and polished using Medaka (v.1.3.0) (https://github.com/nanoporetech/medaka, accessed on 15 May 2021) followed by BLAST-based filtering, keeping contigs matching tick mitochondrial genomes.

##### 2.5.7. Tick Species Confirmation

Species confirmation of the metagenomic characterized ticks was performed using assembled mitochondrial genomes and the animal identification engine provided by BOLD [58], in which the mitochondrial cytochrome oxidase subunit 1 (COI) gene was used.

##### 2.5.8. Phylogenetic Analyses

###### Tick Phylogenies

Assembly of 12S rDNA sequences was performed in the CLC Main Workbench 7 (Qiagen, Aarhus, Denmark). Partial 12S rDNA sequences were aligned using the MAFFT algorithm and compared to sequences available in GenBank [48] using BLASTN (v.2.10.0) [49] and to sequences from morphologically determined reference specimens of multiple species of *Hyalomma*. Maximum likelihood 12S rDNA phylogenies were built in MEGA7 [59], and tick sequences were grouped based on their position in the 12S rDNA phylogenies. See Hoffman et al. 2021 [39] for details.

###### Whole Genome and Mitochondrion Phylogenies

Publicly available sequences for *Francisella*, *Rickettsia*, *Midichloria*, and tick mitochondria, as determined by the GTDB (bacteria) and NCBI (ticks) taxonomies, were downloaded from NCBI (Table S1 in the Supplementary Materials) using NCBI-genome-download (v.0.3.0) (https://github.com/kblin/ncbi-genome-download, accessed on 15 May 2021). Using the workflow manager Snakemake (v.6.2.1) [52], the genome assemblies and the public genomes were aligned pairwise with prograssiveMauve (v.2015_02_13) to selected reference genomes: *F. tularensis tularensis* strain SCHUS4 (GCF_000008985.1) for *Francisella*-positive samples, *Rickettsia rickettsii* strain Iowa (GCA_000017445.3) for *Rickettsia*-positive samples, *Midichloria mitochondrii* strain IricVA (GCA_000219355.1) for *Midichloria*-positive samples, and *Hyalomma asiaticum* strain WY042-2 (NC_053941) for tick mitochondrial sequences. The Python script included in CanSNPer (v.1.0.8) [60] was used to set the alignments to the reference coordinates and to merge them into a multi-FASTA file. IQ-TREE (v.2.1.2) [61] with ModelFinder setting (-m TEST) was used to create the four separate phylogenies. The selected best fit models according to Bayesian Information Criterion (BIC) for *Francisella* was GTR + F + I + G4, for *Rickettsia* TVM + F + I + G4, for *Midichloria* TVM + F + I + G4, and for tick mitochondria K3Pu + F + I + G4. The trees were recalculated with the selected models, and support values were calculated with bootstrap –b 100. The trees were visualized using iTOL [62]. The *Francisella* tree was rooted in Clade 2 according to a previous publication [7], the *Midichloria* and *Rickettsia* phylogenies were rooted in *Orientia tsutsugamushi* (Genome: *Orientia tsutsugamushi* strain Karp GCF_900327275.1) according to the Encyclopedia of Life [63], and the tick mitochondrion phylogeny was rooted by *Rhipicephalus decoloratus* (NC_053941) [64].

### 2.6. Genome Analysis

#### 2.6.1. Average Nucleotide Identity

The similarity between two genomes at the nucleotide-level, average nucleotide identity (ANI), was calculated pairwise for all genomes within each dataset using pyANI (v.0.2.10) with ANIb (BLASTN+) method setting [65].

#### 2.6.2. Biotin Synthesis Pathways

Previous analyses have identified multiple genes involved in the biotin synthesis pathway in the FLE of the tick *O. moubata*, including *bioA*, *bioB*, *bioC*, *bioD*, and *bioF* [11,13]. Homologs to these genes are present in the genome of the FLE of the tick species *Argus arboreus* (*Francisella persica*) [66]. To assess the conservation of these same genes in the genomes of the *Francisella*-like and *Midichloria* endosymbionts from *H. rufipes* samples D14IT15.2 and D14IT20, sequencing reads from both the enriched and non-enriched metagenomic data for these samples were mapped to these genes in the *F. persica* and *M.* *mitochondrii* genomes with minimap2 (v.2.22) [67] and the breadth of coverage was calculated with Samtools (v.1.11) [68] at a minimum depth of 3X.

## 3. Results

### 3.1. Bird Trapping and Tick Collection

In total, 10,209 birds were trapped and screened for ticks. Of these, 244 (2.4%) birds were found to be infested by ticks (*n* = 575) (Table S2 in the Supplementary Materials). Most of the tick-infested birds were long-distance migrants (98.0%). See Hoffman et al. [39] for additional details and information about the distribution pattern of ticks on the bird species.

### 3.2. Tick Determination

**Figure 1:**
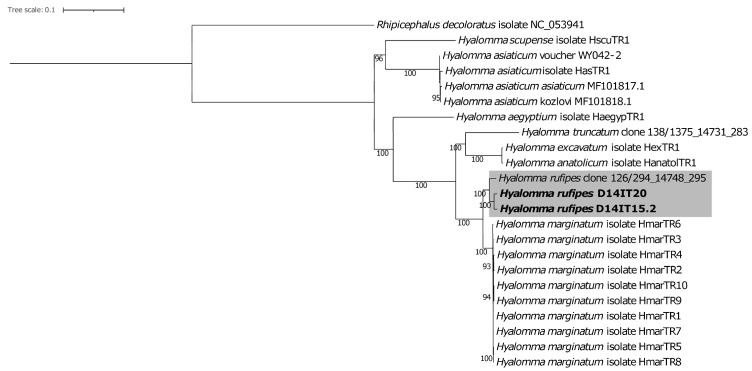
Maximum likelihood phylogeny of *Hyalomma* based on mitochondrial genomes. Study genomes (in bold) form a highly supported clade together with the species *Hyalomma rufipes* (shaded area). *Rhipicephalus decoloratus* was used to root the tree. Bootstrap values ≥ 75 are presented at the nodes. The scale bar represents the expected number of substitutions per site.

The collected ticks were assigned to the Ixodidae genera *Hyalomma*, *Ixodes, Amblyomma*, and *Haemaphysalis*, according to their position in phylogenies based on partial 12S rDNA sequences [39]. The assignment was not possible for 11.5% of the ticks due to the absence of PCR amplicons. The most common tick species were *H. rufipes* and *H. marginatum* [39]. The two metagenomics characterized ticks were confirmed as *H. rufipes* based on their similarity to a known COI sequence from *H. rufipes* and their positioning in the *Hyalomma* 12S rDNA [39] and mitochondrion (Figure 1) phylogenies.

### 3.3. Detection and Determination

#### 3.3.1. Francisella

Results from the microfluidic qPCR suggested the presence of *Francisella* spp. in 72.5% (417/575; *fopA+*) of the total collected ticks, including 77.0% (371/482) of the *Hyalomma* ticks (*H. rufipes*: 76.7% (343/447); *H. marginatum*: 75% (18/24)), 5.9% (1/17) of the *Ixodes* ticks, 100% (2/2) of the *Haemaphysalis* ticks, 62.5% (5/8) of the *Amblyomma* ticks, and 57.6% (38/66) of the undetermined ticks. Furthermore, screening results from the microfluidic qPCR suggested the putative presence of *F. tularensis* in two *H. rufipes* ticks (0.3%; 2/575; *fopA*+ and *lpnA*/Tul4+) collected from two whinchats (*Saxicola rubetra*) trapped on the island of Capri (Italy) in 2014, a result that was not confirmed using the *F. tularensis* specific Tul4 and iQFt1 primers and probes (Table 2).

#### 3.3.2. Spotted Fever Group *Rickettsia*

The microfluidic qPCR data suggested the presence of SFGR in 59.1% (340/575, *gltA*+) of the total collected ticks, including 60.0% (289/482) of *Hyalomma* ticks (*H. rufipes*: 61.5% (275/447); *H. marginatum*: 50.0% (12/24)), 17.6% (3/17) of *Ixodes* ticks, 100% (2/2) of *Haemaphysalis* ticks, 50.0% (4/8) of *Amblyomma* ticks, and 63.6% (42/66) of the undetermined ticks. Presence of SFGR was confirmed in 26 out of 38 analyzed samples by comparison with 17 kDa sequences deposited in GenBank.

#### 3.3.3. Co-Occurrence

Screening data suggested the presence of both *Francisella* and SFGR spp. in 47.1% (271/575; *fopA*+ and *gltA*+) of the total collected ticks, including 48.8% (235/482) of *Hyalomma* ticks (*H. rufipes*: 50.6% (226/447); *H. marginatum*: 29.2% (7/24)), 5.9% (1/17) of *Ixodes* ticks, 100% (2/2) of *Haemaphysalis* ticks, 25% (2/8) of *Amblyomma* ticks, and 47% (31/66) of the undetermined ticks.

#### 3.3.4. Metagenomics

Classification of metagenomic sequencing reads from tick samples that were putatively positive for *F. tularensis* by microfluidic qPCR confirmed the presence of FLEs and not *F. tularensis*. However, assembly of complete *Francisella* genomes was not possible from these data due to low read count and coverage (Table 3). Two rounds of *Francisella* enrichment were therefore performed, resulting in >95% *Francisella* DNA after enrichment. The assembly of *Francisella* DNA present in the sequenced enriched sample D14IT15.2 was in total 1,413,985 bp divided into 510 contigs with N50 = 10,189 and N50n = 36. The assembly of *Francisella* DNA present in the sequenced enriched sample D14IT20 was, in total, 1,415,455 bp divided into 506 contigs with N50 = 11,392 and N50n = 35.

The levels of *Rickettsia* and *Midichloria* DNA present in the metagenomic data were relatively high compared to that of *Francisella* DNA. The assembly of *Rickettsia* DNA present in the sequenced non-enriched sample D14IT15.2 was in total 1,317,746 bp divided into 20 contigs with N50 = 225,990 and N50n = 3. The assembly of *Rickettsia* DNA present in the sequenced non-enriched sample D14IT20 was in total 1,318,206 bp divided into 20 contigs with N50 = 225,819 and N50n = 3. The assembly of *Midichloria* DNA present in the sequenced non-enriched sample D14IT15.2 was in total 1,069,525 bp divided into 105 contigs with N50 = 17,240 and N50n = 23. The assembly of *Midichloria* DNA present in the sequenced non-enriched sample D14IT20 was in total 952,239 bp divided into 151 contigs with N50 = 9933 and N50n = 34.

#### 3.3.5. Phylogenetic Inference of Metagenome-Assembled Genomes

**Figure 2:**
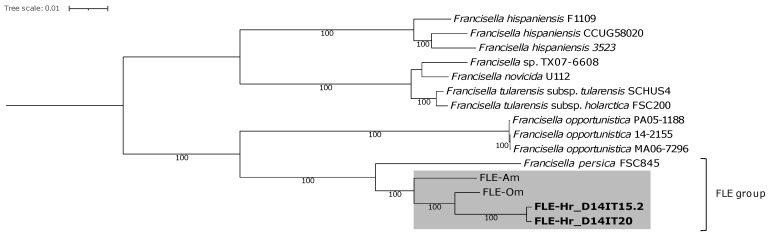
Whole-genome maximum likelihood phylogeny of Clade 1 *Francisella*. The phylogeny contains 13 representative *Francisella* genomes, and the shaded area indicates the subclade within the FLE group (GTDB cluster: *Francisella* sp002095075) in which the *Francisella* metagenome-assembled genomes (in bold) generated in this study are assigned. The tree was rooted in Clade 2 of *Francisella* (not shown). Bootstrap values ≥ 75 are presented at the nodes. The scale bar represents the expected number of substitutions per site. FLE*—Francisella*-like endosymbiont; FLE-Om (host: *Ornithodoros moubata*); FLE-Am (host: *Amblyomma maculatum*); FLE-Hr (detected in *Hyalomma rufipes*); sp.—species; subsp.—subspecies.

Phylogenetic inference of the MAGs of *Francisella* revealed that D14IT15.2 and D14IT20 belonged to Clade 1 of *Francisella* [7], are separated from *F. tularensis* (FSC200, SCHUS4), and are members of a subclade within the FLE group (GTDB cluster: *Francisella* sp002095075) (Figure 2, shaded area). The FLE group consists of both cluster *F. persica* and *Francisella* sp002095075. Characterization of metagenome-assembled *Midichloria* and *Rickettsia* genomes revealed resemblance to *M. mitochondrii* and *R. aeschlimannii*, respectively, with the latter within the clade of *Rickettsia rhipicephali*, according to the taxonomy used by GTDB (Figures S1 and S2 in the Supplementary Materials).

### 3.4. Genome Analyses

#### 3.4.1. Average Nucleotide Identity

The highest ANI values observed were between the generated MAGs and the genomes of FLE-Om, *R. rhipicephali* (*R. aeschlimannii*), *M. mitochondrii*, and *H. rufipes*, respectively (Table 4, Figures S3–S6 in the Supplementary Materials). In GTDB *R. aeschlimannii* belongs to the species *R. rhipicephali* while it is a recognised species in NCBI. The sequence identity between the two genomes of: (i) FLE-Hr (FLE_D14IT15.2 and FLE_D14IT20) was 99.7–99.8%, (ii) *Midichloria*-Hr (MID_D14IT15.2 and MID_D14IT20) 99.9–100%, (iii) *Rickettsia*-Hr (RICK_D14IT15.2 and RICK_D14IT20) 100%, and (iv) *Hyalomma* (HYA_D14IT15.2 and HYA_D14IT20) 99.3%.

#### 3.4.2. Biotin Gene Conservation

Based upon the mapping of reads to the corresponding *F. persica* coding DNA sequences (CDSs) encoding homologs to *bioA*, *bioB*, *bioC*, *bioD*, and *bioF*, at least *bioA* appears to be missing in the FLE MAGs generated from the enrichment of samples D14IT15.2 and D14IT20, indicating the biotin pathway is not intact in these FLEs (Table 5). We note that mapping of FLE reads from the non-enriched metagenomic sequencing data to these same CDSs was very limited, owing to the low proportion of FLE reads in these data (Table 3). In contrast, there was significant mapping of reads from the non-enriched metagenomic data to all of the examined biotin genes in the *M. mitochondrii* genome, indicating that this pathway is likely intact in the *Midichloria* endosymbiont of *H. rufipes*. There was a limited mapping of reads from the enriched metagenomic data to the biotin genes in *M. mitochondrii*, which is not unexpected given the high proportion of *Francisella* DNA in these samples following enrichment.

## 4. Discussion

In this study, screening of ticks infesting birds migrating in a northward route from wintering areas in Africa revealed a high prevalence of *Francisella* in the tick species *H. rufipes* (*fopA*+: 76.7%), a known vector of SFGR and Crimean-Congo hemorrhagic fever virus [69], which suggest that migratory birds in the AWPR may contribute to northward dispersal of *Francisella*-infected ticks. The high prevalence of *Francisella* likely represents a high prevalence of FLEs, as FLEs have previously been detected in multiple species of *Hyalomma* ticks and at a high prevalence [17,20,70]. The two *Francisella* MAGs generated from two *H. rufipes* (Hr) were found to be members of a subclade within the FLE group (GTDB cluster: *Francisella* sp002095075) in Clade 1 of *Francisella* (Figure 2), which includes the FLE species FLE-Om (present in the soft tick *O. moubata*) and FLE-Am (present in the hard tick *A. maculatum*). The FLE-Hr had the highest identity to FLE-Om (ANIb: 96.7–97.0%). Several ticks tested positive for both *Francisella* and SFGR spp. (*fopA*+/*gltA*+: 47.1%), suggesting that presence of *Francisella* did not prevent the occurrence of SFGR spp.; a similar observation has been reported by Scoles [19]. ANI and phylogenetic inference indicated the highest similarity of the *Rickettsia* MAGs detected in *H. rufipes* (*Rickettsia*-Hr) with *R. aeschlimannii* (ANIb: 98.8–99.9%), a SFGR (i) reported to cause human infections [71,72], (ii) associated with *Hyalomma* ticks, including *H. rufipes* and *H. marginatum* [34,73,74,75,76,77], (iii) previously identified together with FLEs in *H. marginatum* [23], and (iv) detected at similar prevalences as in this study in ticks of the *H. marginatum* species complex infesting northbound migratory birds [36,78]. Data from the two metagenomic sequenced *H. rufipes* ticks indicated co-occurrence also with a species of *Midichloria*. ANI and phylogenetic inference of the *Midichloria* MAGs detected in *H. rufipes* (*Midichloria*-Hr) indicated a close relationship to *M. mitochondrii* (ANIb: 91.5–92.3%). *Midichloria* sp. bacteria have previously been detected in *H. marginatum* species complex ticks infesting northbound trans-Saharan spring migrating birds trapped in Italy [47]. That study found a high prevalence of *Midichloria* DNA in the investigated *Hyalomma* ticks (>90%) and in a considerable fraction of the blood samples from the avian hosts (>40%) and suggested that the presence of *Midichloria* DNA in the blood was associated with lower fat reserves in the tick-infested birds [47].

Ticks may depend on FLEs because they provide nutrients that are absent in the tick diet, such as B vitamins (folate/folic acid (B_9_), riboflavin (B_2_), and biotin (B_7_)) and co-factors, and thereby improve the fitness of the tick [5,11]. It has been suggested that FLEs serve as alternative obligate symbionts in some species of ticks [5,79], whereas *Coxiella*-like endosymbionts (CLEs) are considered to be obligate symbionts (i.e., present in most specimens) in most tick species [6,80]. Ticks may escape a negative symbiosis by replacing an old symbiont with a new bacterium [81]. FLEs may have replaced CLEs in several tick lineages, including *O. moubata* and *A. maculatum* [5,13,79]. *M. mitochondrii* has also been suggested to be a nutritional endosymbiont since it encodes genes for the production of several co-factors and B vitamin biotin [82]. Buysse et al. [23] showed that the genomes of FLEs detected in *H. marginatum* included functional biosynthesis pathways for folate and riboflavin but were deprived of a functional biosynthesis pathway for biotin. The authors suggested that this was compensated for by the co-symbiosis with *Midichloria* bacteria also present in *H. marginatum*, since their genomes included an intact biotin biosynthesis operon [23]. The *Midichloria* detected in *H. marginatum* had a partial riboflavin biosynthesis pathway, indicating that co-occurrence of FLEs and *Midichloria* may be essential for complete nutritional symbiosis in *H. marginatum* [23]. We observed similar patterns for *H. rufipes*: a disrupted biotin biosynthesis pathway in its FLE but an apparently intact biotin biosynthesis pathway within its *Midichloria* endosymbiont (Table 5), suggesting the co-occurrence of these bacteria also may be essential for nutritional symbiosis in *H. rufipes*. As noted by Buysse et al. [23], a similar dual symbiosis may be present in several other *Hyalomma* tick species (*Hyalomma aegyptium*, *Hyalomma anatolicum*, *Hyalomma dromedarii*, *Hyalomma excavatum*, *Hyalomma impeltatum*, *Hyalomma lusitanicum*, and *Hyalomma truncatum*), as they found evidence for the presence of both FLEs and *Midichloria* within them. The apparent exception within this tick genus to date appears to be *Hyalomma asiaticum*, which does not harbor any *Midichloria* but instead has an FLE with an intact biotin pathway [83].

Molecular species determination of members of the *H. marginatum* species complex—currently consisting of five species, including *H. marginatum* and *H. rufipes* [84]—can be difficult due to the inclusion in public databases of sequences obtained from incorrectly identified specimens [46]. Complete tick mitochondrial MAGs were therefore constructed to verify the initial 12S rDNA-based speciation of the metagenomically characterized ticks. The two characterized tick specimens were found to group in the *H. rufipes* clade also in the mitochondrion phylogeny, verifying the initial 12S rDNA speciation results.

## 5. Conclusions

Understanding the biology, ecology, and evolution of tick endosymbionts is important, as they may share a close evolutionary relationship with pathogenic bacteria and also may influence the fitness [4] and even the behavior of the tick host [85]. The results of this study demonstrate that FLEs are present in many *H. rufipes* ticks, and migratory birds in the AWPR contribute to the northward geographical spread of FLE-containing ticks. The absence of *F. tularensis* in the investigated ticks does not provide evidence supporting that immature life stages of *H. rufipes* contribute to the transmission of *F. tularensis* in the study region. Furthermore, the results suggest that migratory birds also contribute to northward geographical spread in the AWPR of *H. rufipes* ticks containing SFGR spp., including *R. aeschlimannii* and *Midichloria* bacteria, and that a dual endosymbiosis (co-symbiosis) of FLEs and *Midichloria* may support the nutritional requirements of the medically important tick vector *H. rufipes.* We acknowledge that the majority of the results of this study are based on unconfirmed screening data and that the reported detection results are therefore conservative estimates of the prevalence. Future studies should therefore focus on verifying the *Francisella* and SFGR prevalence in *H. rufipes* as well as investigate the *Midichloria* prevalence in *H. rufipes* and the impact that FLEs and *Midichloria* may have on *H. rufipes*, including their interaction with bacterial pathogens, such as SFGR.

## Figures and Tables

**Table 1:** Primers and probes used for real-time PCR assays targeting tick taxa, *Francisella*, and *Rickettsia*.

Organism	Genus/Group/ Species	Target Gene	PCR ID	Name	Sequence (5′ → 3′)	Amplicon (bp)	Reference
Tick		12S rDNA	12S	T1B	AAA CTA GGA TTA GAT ACC CT	320	[43]
				T2A	AAT GAG AGC GAC GGG CGA TGT		
*Francisella*		*fopA*		Forward	GGC AAA TCT AGC AGG TCA AGC		[40]
	*Francisella*		FopA	Reverse	CAA CAC TTG CTT GAA CAT TTC TAG	89	
				Probe	GGT GCT TGG GAT GTG GGT GGT G		
		*sucC*		Forward	AAC TGG CTG ACC TTC AGC AT		[41]
	*Francisella*		GF1	Reverse	GTG GTC GTG GTA AAG CTG GT	125	
				Probe	CCG ATT AGG CTT TCT GCT ACT TCA CGA		
		*lpnA*	Tul4	Forward	ACC CAC AAG GAA GTG TAA GAT TA	76	[40]
	*F. tularensis*			Reverse	GTA ATT GGG AAG CTT GTA TCA TG		
				Probe	AAT GGC AGG CTC CAG AAG GTT CTA AGT		
				Forward	CGC AGG TTT AGC GAG CTG TT		
	*F. tularensis*	*lpnA*	iQFt1	Reverse	GCA GCT TGC TCA GTA GTA GCT GTC T	108	[42]
				Probe	CAT CAT CAG AGC CAC CTA ACC CTA		
*Rickettsia*	SFG	*gltA*	GltA	SFG_gltA_F	CCT TTT GTA GCT CTT CTC ATC C	145	[40]
				SFG_gltA_R	GCG ATG GTA GGT ATC TTA GCA A		
				SFG_gltA_P	TGG CTA TTA TGC TTG CGG CTG TCG GT		
	*Rickettsia*	17 kDa	17 kDa	Rr17 kDa.61p	GCT CTT GCA AC TTC TAT GTT	434	[45]
				Rr17 kDa.492n	CAT TGT TCG TCA GGT TGG CG		

bp—base pairs; 12S rDNA—12S ribosomal DNA gene; *fopA*—gene encoding the outer membrane protein A (FopA); *sucC*—gene encoding the succinyl-CoA ligase [ADP-forming] subunit beta; *lpnA*—gene encoding the lipoprotein A (LpnA); *gltA*—gene encoding citrate synthase; SFG—spotted fever group; 17 kDa—17 kilo Dalton surface antigen.

**Table 2:** Confirmation results of the two ticks that tested positive for the putative presence of *Francisella tularensis* DNA during screening.

Method	Microfluidic qPCR (Screening)		qPCR (Confirmation)			
Species/Genus	*F. tularensis*	*Francisella*	*F. tularensis*		*Francisella*	
Tick/PCR ID	Tul4	FopA	iQFt1	Tul4	GF1	FopA
D14IT15.2	Positive Ct = 26.9	Positive Ct = 22.3	Negative Ct = N/A	Negative Ct = N/A	Positive Ct = 28.2	Positive Ct = 29.6
D14IT20	Positive Ct = 26.6	Positive Ct = 21.6	Negative Ct = N/A	Negative Ct = N/A	Positive Ct = 19.8	Positive Ct = 29.6

PCR—polymerase chain reaction; qPCR—real-time PCR; Ct—cycle threshold value; N/A—not available.

**Table 3:** Relative abundance of bacterial reads of selected species in metagenomic sequence data according to Kraken 2 results for the two ticks testing putatively positive for presence of *Francisella tularensis* by microfluidic real-time PCR. Species determination according to the Genome Taxonomy Database.

Tick ID	D14IT15.2		D14IT20	
**Total Reads (M)**	225.9		173.6	
**Genus/Species**	**Reads (M)**	**%**	**Reads (M)**	**%**
*Rickettsia*	44.4	19.7	103.5	59.6
*R. rhipicephali*	38.8	17.2	93.2	53.7
*Midichloria*	9.4	4.2	0.95	0.55
*M. mitochondrii*	9.4	4.2	0.95	0.55
*Francisella*	0.080	0.0035	0.026	0.015
FLE	0.074	0.0035	0.025	0.014

M—million; FLE—*Francisella*-like endosymbiont.

**Table 4:** Highest average nucleotide identity for bacterial (*n* = 6) and tick (*n* = 2) metagenome-assembled genomes generated in this study detected in two avian-associated *Hyalomma rufipes* ticks (D14IT15.2 and D14IT20) that tested positive for *Francisella tularensis* and spotted fever group *Rickettsia* during screening.

Tick ID	D14IT15.2		D14IT20	
Organism	ANIb (%)	Genome	ANIb (%)	Genome
FLE-Om	96.8/97.0	MAG	96.7/96.9	MAG
(GCF_002095075)				
*Rickettsia rhipicephali*^1^ (*R. aeschlimannii* ^2^)	99.8/99.8	MAG	99.8/99.9	MAG
(GCA_001051325)				
*Midichloria mitochondrii*	91.5/91.7	MAG	91.8/92.3	MAG
(GCA_000219355)				
*Hyalomma rufipes*	98.1/98.1	MAG ^3^	98.0/98.1	MAG ^3^
(KY457528)				

^1^ According to the Genome Taxonomy Database; ^2^ According to the National Center for Biotechnology Information; ^3^ Mitochondrion; MAG—metagenome-assembled genome; ANIb—average nucleotide identity, BLAST method; FLE—*Francisella*-like endosymbiont; Om—*Ornithodoros moubata*.

**Table 5:** Coverage breadth (%) of sequencing reads of samples D14IT15.2 and D14IT20 mapped to genes involved in the biotin synthesis in the genome of *Francisella persica* and *Midichloria* *mitochondrii*, indicating an intact biotin synthesis pathway of *Midichloria* and a disrupted biotin synthesis pathway of *Francisella*-like endosymbionts detected in the tick species *Hyalomma rufipes*.

	Enriched		Non-Enriched		
Homolog in *Midichloria*/*Francisella* Genomes	D14IT15.2	D14IT20	D14IT15.2	D14IT20	Genes Involved in the Biotin Synthesis
lcl|NC_015722.1_cds_WP_013950979.1_473 [*M. mitochondrii*]	0 *	0	100	100	*bioA*
lcl|NC_015722.1_cds_WP_013950663.1_135 [*M. mitochondrii*]	13.4	0	99.0	98.6	*bioB*
lcl|NC_015722.1_cds_WP_237697388.1_131 [*M. mitochondrii*]	0	0	99.7	95.4	*bioC*
lcl|NC_015722.1_cds_WP_013950658.1_130 [*M. mitochondrii*]	0	0	93.7	93.5	*bioD*
lcl|NC_015722.1_cds_WP_237697389.1_134 [*M. mitochondrii*]	0	0	88.0	87.9	*bioF*
lcl|NZ_CP013022.1_cds_WP_064461154.1_1224 [*F. persica*]	21.5	19.7	0	0	Homolog to *bioA* in *F. tularensis* (FTT_0938)
lcl|NZ_CP013022.1_cds_WP_064461748.1_1225 [*F. persica*]	100	100	0	0	Homolog to *bioB* in *F. tularensis* (FTT_0937c)
lcl|NZ_CP013022.1_cds_WP_064461156.1_1227 [*F. persica*]	76.3	74.3	0	0	Homolog to *bioC* in *F. tularensis* (FTT_0935c)
lcl|NZ_CP013022.1_cds_WP_064461157.1_1228 [*F. persica*]	99.3	99.3	0	32.5	Homolog to *bioD* in *F. tularensis* (FTT_0934c)
lcl|NZ_CP013022.1_cds_WP_064461155.1_1226 [*F. persica*]	63.3	64.4	0	0	Homolog to *bioF* in *F. tularensis* (FTT_0936c)
*Reads deposited to NCBI sequence read archive*	*SRR16203935*	*SRR16203936*	*SRR16203939*	*SRR16203940*	

* Values are color-coded and the highest values are indicated by bright red.

## Data Availability

The metagenomic sequence data generated in this study are available under the NCBI BioProjectPRJNA764565.

## Supplementary Materials

Supporting information can be downloaded at: https://www.mdpi.com/article/10.3390/microorganisms10071393/s1, Table S1. Publicly available genomes for *Francisella, Rickettsia, Midichloria*, and tick mitochondria downloaded from GTDB and NCBI. Table S2. Microfluidic real-time PCR data for ticks collected from bird species trapped in the Mediterranean basin during the spring migration of 2014 and 2015. The ticks were screened for *Francisella* and spotted fever group *Rickettsia* species using primers targeting the *fopA* and *gltA* genes. Figure S1. Whole genome maximum likelihood phylogeny. Highlighted area indicates *Midichloria* clade. Study genomes are in bold. *Orientia tsutsugamushi* strain Karp was used to root the tree. Bootstrap values ≥ 75 are presented at the nodes. The scale bar represents the expected number of substitutions per site. Figure S2. Whole genome maximum likelihood phylogeny of *Rickettsia*. Highlighted area indicates clade for *Rickettsia aeschlimannii*. Study genomes are in bold. *Orientia tsutsugamushi* was used to root the tree. Bootstrap values ≥ 75 are presented at the nodes. The scale bar represents the expected number of substitutions per site. Figure S3. Heatmap of the average nucleotide identity (ANI), demonstrating nucleotide-level genomic similarity between *Francisella* genomes. The pairwise comparison of 19 *Francisella* genomes was computed by BLAST, using the pyANI software. Study genomes are in bold. FLE, *Francisella*-like endosymbiont. Figure S4. Heatmap of the average nucleotide identity (ANI), demonstrating nucleotide-level genomic similarity between *Rickettsia* genomes. The pairwise comparison of 138 *Rickettsia* genomes was computed by BLAST, using the pyANI software. Study genomes are in bold. For NCBI organism names, see Table S1. Figure S5. Heatmap of the average nucleotide identity (ANI), demonstrating nucleotide-level genomic similarity between bacterial genomes. The pairwise comparison of 23 genomes was computed by BLAST, using the pyANI software. Study genomes are in bold. Figure S6. Heatmap of the average nucleotide identity (ANI), demonstrating nucleotide-level genomic similarity between *Hyalomma* mitochondrial genomes. The pairwise comparison of 23 *Hyalomma* mitochondrial genomes was computed by BLAST, using the pyANI software. Study genomes are in bold.
