# Supplementary material for: Co-Occurrence of Francisella, Spotted Fever Group Rickettsia, and Midichloria in Avian-Associated Hyalomma rufipes
Source: Microorganisms. 2022 Jul 11;10(7):1393. doi: 10.3390/microorganisms10071393 (PMC9323704; doi:10.3390/microorganisms10071393)
Supplement: Supplementary file 1 [file microorganisms-10-01393-s001.zip › microorganisms-1693673-supplementary.pdf]

Supplementary Materials

Tables

Table S1. Publicly available genomes for *Francisella*, *Rickettsia*, *Midichloria,* and tick mitochondria downloaded from GTDB and NCBI.

Taxa	Genbank assembly assession	NCBI organism name	GTDB taxonomy (Release 06-RS202)
Francisella	GCF_000168775	*Francisella tularensis* subsp. *holarctica* str. FSC200	s__Francisella tularensis
	GCF_000008985	*Francisella tularensis* subsp. *tularensis* str. SCHUS4	s__Francisella tularensis
	GCF_000014645	*Francisella novicida* str. U112	s__Francisella tularensis
	GCF_001870885	*Francisella* sp. str. TX07-6608	s__Francisella tularensis
	GCF_016604405	*Francisella hispaniensis* str. F1109	
	GCF_001608445	*Francisella hispaniensis* str. CCUG 58020	s__Francisella hispaniensis
	GCF_000195555	*Francisella hispaniensis* str. 3523	s__Francisella hispaniensis
	GCF_003347135	*Francisella opportunistica* str. PA05-1188	s__Francisella opportunistica
	GCF_003347095	*Francisella opportunistica* str. 14-2155	s__Francisella opportunistica
	GCF_003347115	*Francisella opportunistica* str. MA06-7296	s__Francisella opportunistica
	GCF_001275365	*Francisella persica* ATCC VR-331 str. FSC845	s__Francisella persica
	GCA_001753795	*Francisella* endosymbiont of *Amblyomma maculatum* str. FLE-Am	s__Francisella sp002095075
	GCF_002095075	*Francisella* endosymbiont of *Ornithodoros moub*ata str. FLE-Om	s__Francisella sp002095075
	GCF_000195535	*Francisella cf. novicida* Fx1	s__Francisella tularensis
	GCF_001865695	*Francisella tularensis* subsp. *novicida* PA10-7858	s__Francisella tularensis
	GCA_016604535	*Francisella tularensis* subsp. *novicida* FSC595	
	GCF_000833355	*Francisella tularensis* subsp. *novicida* D9876	s__Francisella tularensis
	GCF_000154265	*Francisella tularensis* subsp. *novicida* GA99-3548	s__Francisella tularensis
	GCA_016604595	*Francisella tularensis* subsp. *novicida* FSC159	
Midichloria	GCA_000219355	*Candidatus* Midichloria mitochondrii str. IricVA	g__Midichloria
	GCA_000730245	Endosymbiont of *Acanthamoeba* sp. UWC8	g__Jidaibacter
	GCA_000815465	*Candidatus* Jidaibacter acanthamoeba sp. UWC36	g__Jidaibacter
	GCA_003072485	*Candidatus* Fokinia solitaria	g__Fokinia
	GCA_003953955	*Candidatus* Aquarickettsia rohweri	g__Aquarickettsia
	GCA_004210275	Rickettsiales endosymbiont of *Peranema trichophorum*	g__RICK02
	GCA_009690945	Rickettsiales endosymbiont of Trichoplax	g__Aquarickettsia
	GCA_013288625	Alphaproteobacteria bacterium	g__JABDBN01
	GCA_014116815	Holosporaceae bacterium str. Namur	g__Jidaibacter
	GCA_903820055	uncultured Alphaproteobacteria bacterium	g__Aquarickettsia
	GCA_903860855	uncultured Alphaproteobacteria bacterium	g__Aquarickettsia
	GCA_903865125	uncultured Alphaproteobacteria bacterium	g__CAIOSF01
	GCA_903878005	uncultured Alphaproteobacteria bacterium	g__CAIOSF01
	GCA_903887235	uncultured Alphaproteobacteria bacterium	g__Aquarickettsia
	GCA_903903795	uncultured Alphaproteobacteria bacterium	g__Aquarickettsia
	GCA_903916125	uncultured Alphaproteobacteria bacterium	g__Aquarickettsia
	GCA_903926815	uncultured Alphaproteobacteria bacterium	g__Aquarickettsia
	GCA_903953855	uncultured Alphaproteobacteria bacterium	g__Aquarickettsia
	GCA_903957775	uncultured Alphaproteobacteria bacterium	g__Aquarickettsia
	GCA_905479795	uncultured *Candidatus* Midichloriaceae bacterium	
Rickettsia	GCF_900327275	*Orienta tsutsugamushi* str. Karp	s__Orientia tsutsugamushi
	GCF_016743795	*Rickettsia* sp. str. Tillamook 23	
	GCA_000012145	*Rickettsia felis* str. URRWXCal2	s__Rickettsia felis
	GCA_000385475	*Rickettsia prowazekii* str. GvF12	s__Rickettsia prowazekii
	GCA_000696365	*Rickettsia buchneri* str. ISO7	s__Rickettsia buchneri
	GCA_000751075	*Rickettsia tamurae* str. AT-1	s__Rickettsia buchneri
	GCA_000964845	*Rickettsia hoogstraalii* str. RCCE3	s__Rickettsia hoogstraalii
	GCA_001051325	*Rickettsia aeschlimannii*	s__Rickettsia rhipicephali
	GCA_003664375	*Candidatus* Rickettsia colombianensi str. Adcor 2	s__Rickettsia buchneri
	GCF_000007025	*Rickettsia conorii* str. Malish 7	s__Rickettsia rickettsii
	GCF_000008045	*Rickettsia typhi* str. Wilmington	s__Rickettsia typhi
	GCF_000012385	*Rickettsia bellii* str. RML369-C	s__Rickettsia bellii
	GCF_000014345	*Rickettsia canadensis* str. McKiel	s__Rickettsia canadensis
	GCF_000016625	*Rickettsia massiliae* str. MTU5	s__Rickettsia rhipicephali
	GCF_000017445	*Rickettsia rickettsii* str. Iowa	s__Rickettsia rickettsii

	GCF_000018205	*Rickettsia akari* str. Hartford	s__Rickettsia akari
	GCF_000018225	*Rickettsia rickettsii* str. Sheila Smith	s__Rickettsia rickettsii
	GCF_000018245	*Rickettsia bellii* str. OSU 85-389	s__Rickettsia bellii
	GCF_000021525	*Rickettsia peacockii* str. Rustic	s__Rickettsia rickettsii
	GCF_000022785	*Rickettsia prowazekii* str. Rp22	s__Rickettsia prowazekii
	GCF_000023005	*Rickettsia africae* str. ESF-5	s__Rickettsia rickettsii
	GCF_000160735	*Rickettsia* endosymbiont of *Ixodes scapularis*	s__Rickettsia buchneri
	GCF_000166935	*Rickettsia sibirica* str. 246	s__Rickettsia rickettsii
	GCF_000195735	*Rickettsia prowazekii* str. Madrid E	s__Rickettsia prowazekii
	GCF_000221205	*Rickettsia heilongjiangensis* str. 054	s__Rickettsia rickettsii
	GCF_000237845	*Rickettsia slovaca* str. 13-B	s__Rickettsia rickettsii
	GCF_000246715	*Rickettsia sibirica* subsp. *sibirica* str. BJ-90	s__Rickettsia rickettsii
	GCF_000247625	*Rickettsia sibirica* subsp. *mongolitimonae* str. HA-91	s__Rickettsia rickettsii
	GCF_000252365	*Rickettsia slovaca* str. D-CWPP	s__Rickettsia rickettsii
	GCF_000255355	*Rickettsia helvetica* str. C9P9	s__Rickettsia helvetica_A
	GCF_000257435	*Rickettsia conorii* subsp. *indica* str. ITTR	s__Rickettsia rickettsii
	GCF_000261325	*Rickettsia conorii* subsp. *caspia* str. A-167	s__Rickettsia rickettsii
	GCF_000263055	*Rickettsia honei* str. RB	s__Rickettsia rickettsii
	GCF_000263815	*Rickettsia conorii* subsp. *israelensis* str. ISTT CDC1	s__Rickettsia rickettsii
	GCF_000265225	*Rickettsia* sp. str. MEAM1 (*Bemisia tabaci*)	s__Rickettsia sp002285905
	GCF_000273745	*Rickettsia australis* str. Phillips	s__Rickettsia australis
	GCF_000277165	*Rickettsia prowazekii* str. Chernikova	s__Rickettsia prowazekii
	GCF_000277185	*Rickettsia prowazekii* str. Katsinyian	s__Rickettsia prowazekii
	GCF_000277205	*Rickettsia prowazekii* str. BuV67-CWPP	s__Rickettsia prowazekii
	GCF_000277225	*Rickettsia prowazekii* str. Dachau	s__Rickettsia prowazekii
	GCF_000277245	*Rickettsia prowazekii* str. GvV257	s__Rickettsia prowazekii
	GCF_000277265	*Rickettsia prowazekii* str. RpGvF24	s__Rickettsia prowazekii
	GCF_000277285	*Rickettsia typhi* str. TH1527	s__Rickettsia typhi
	GCF_000277305	*Rickettsia typhi* str. B9991CWPP	s__Rickettsia typhi
	GCF_000283595	*Rickettsia japonica* str. YH	s__Rickettsia rickettsii
	GCF_000283775	*Rickettsia rickettsii* str. Colombia	s__Rickettsia rickettsii
	GCF_000283795	*Rickettsia rickettsii* str. Arizona	s__Rickettsia rickettsii
	GCF_000283815	*Rickettsia rickettsii* str. Hino	s__Rickettsia rickettsii
	GCF_000283835	*Rickettsia rickettsii* str. Hauke	s__Rickettsia rickettsii
	GCF_000283855	*Rickettsia massiliae* str. AZT80	s__Rickettsia rhipicephali
	GCF_000283915	*Rickettsia canadensis* str. CA410	s__Rickettsia canadensis
	GCF_000283935	*Rickettsia rickettsii* str. Hlp#2	s__Rickettsia rickettsii
	GCF_000283955	*Rickettsia rickettsii* str. Brazil	s__Rickettsia rickettsii
	GCF_000283995	*Rickettsia philipii* str. 364D	s__Rickettsia rickettsii
	GCF_000284055	*Rickettsia amblyommatis* str. GAT-30V	s__Rickettsia rhipicephali
	GCF_000284075	*Rickettsia rhipicephali* str. 3-7-female6-CWPP	s__Rickettsia rhipicephali
	GCF_000284155	*Rickettsia australis* str. Cutlack	s__Rickettsia australis
	GCF_000284175	*Rickettsia montanensis* str. OSU 85-930	s__Rickettsia rhipicephali
	GCF_000284195	*Rickettsia parkeri* str. Portsmouth	s__Rickettsia rickettsii
	GCF_000302635	*Rickettsia japonica* str. YH	s__Rickettsia rickettsii
	GCF_000363905	*Rickettsia prowazekii* str. NMRC Madrid E	s__Rickettsia prowazekii
	GCF_000367405	*Rickettsia prowazekii* str. Breinl	s__Rickettsia prowazekii
	GCF_000385495	*Rickettsia prowazekii* str. Cairo 3	s__Rickettsia prowazekii
	GCF_000485845	*Rickettsia gravesii* str. BWI-1	s__Rickettsia rhipicephali
	GCF_000499665	*Rickettsia monacensis* str. IrR/Munich	s__Rickettsia buchneri
	GCF_000804505	*Rickettsia felis* str. LSU-Lb	s__Rickettsia felis
	GCF_000804525	*Rickettsia felis* str. LSU	s__Rickettsia felis
	GCF_000825685	*Rickettsia hoogstraalii* str. Croatica	s__Rickettsia hoogstraalii
	GCF_000828125	*Rickettsia asembonensis* str. NMRii	s__Rickettsia asembonensis
	GCF_000831525	*Rickettsia rickettsii* str. R	s__Rickettsia rickettsii
	GCF_000831545	*Rickettsia rickettsii* str. Morgan	s__Rickettsia rickettsii
	GCF_000940955	*Rickettsia raoultii* str. Khabarovsk	s__Rickettsia rhipicephali
	GCF_000964665	*Rickettsia felis* str. Pedreira	s__Rickettsia felis
	GCF_000964675	*Rickettsia amblyommatis* str. Ac/Pa	s__Rickettsia rhipicephali
	GCF_000964905	*Rickettsia rhipicephali* str. Ect	s__Rickettsia rhipicephali
	GCF_000964995	*Rickettsia amblyommatis* str. Darkwater	s__Rickettsia rhipicephali
	GCF_000965005	*Rickettsia bellii* str. RML An4	s__Rickettsia bellii
	GCF_000965045	*Rickettsia bellii* str. RML Mogi	s__Rickettsia bellii
	GCF_000965075	*Rickettsia parkeri* str. AT#24	s__Rickettsia rickettsii

	GCF_000965085	*Rickettsia parkeri* str. Grand Bay	s__Rickettsia rickettsii
	GCF_000965145	*Rickettsia parkeri* str. Tate's Hell	s__Rickettsia rickettsii
	GCF_000965155	*Rickettsia* endosymbiont of *Ixodes pacificus* str. Humboldt	s__Rickettsia buchneri
	GCF_000965185	*Rickettsia argasii* str. T170-B	s__Rickettsia rickettsii
	GCF_001273795	*Rickettsia amblyommatis* str. Ac37	s__Rickettsia rhipicephali
	GCF_001442475	*Rickettsia rhipicephali* str. HJ#5	s__Rickettsia rhipicephali
	GCF_001602215	*Rickettsia prowazekii* str. Naples-1	s__Rickettsia prowazekii
	GCF_001602635	*Rickettsia* endosymbiont of *Proechinophthirus fluctus* str. SPI-2	s__Rickettsia rickettsii
	GCF_001653015	*Rickettsia* sp. str. Tenjiku01	s__Rickettsia rickettsii
	GCF_001707805	*Rickettsia* sp. str. wq	s__Rickettsia sp002285905
	GCF_001707925	*Rickettsia* sp. str. wb	s__Rickettsia sp002285905
	GCF_001950995	*Rickettsia rickettsii* str. Iowa	s__Rickettsia rickettsii
	GCF_001951015	*Rickettsia rickettsii* str. Iowa	s__Rickettsia rickettsii
	GCF_001975185	*Rickettsia raoultii* str. IM16	s__Rickettsia rhipicephali
	GCF_002078315	*Rickettsia bellii*	s__Rickettsia bellii
	GCF_002078335	*Rickettsia amblyommatis*	s__Rickettsia rhipicephali
	GCF_002285905	*Rickettsia* sp. str. MEAM1 (*Bemisia tabaci*)	s__Rickettsia sp002285905
	GCF_002356695	*Rickettsia japonica* str. YH_M	s__Rickettsia rickettsii
	GCF_002356715	*Rickettsia japonica* str. SR1567	s__Rickettsia rickettsii
	GCF_002356735	*Rickettsia japonica* str. M99123	s__Rickettsia rickettsii
	GCF_002356755	*Rickettsia japonica* str. M99023	s__Rickettsia rickettsii
	GCF_002356775	*Rickettsia japonica* str. M99015	s__Rickettsia rickettsii
	GCF_002356795	*Rickettsia japonica* str. M14024	s__Rickettsia rickettsii
	GCF_002356815	*Rickettsia japonica* str. M14012	s__Rickettsia rickettsii
	GCF_002356835	*Rickettsia japonica* str. M13010	s__Rickettsia rickettsii
	GCF_002356855	*Rickettsia japonica* str. M11012	s__Rickettsia rickettsii
	GCF_002356875	*Rickettsia japonica* str. M08024	s__Rickettsia rickettsii
	GCF_002356895	*Rickettsia japonica* str. M00021	s__Rickettsia rickettsii
	GCF_002356915	*Rickettsia japonica* str. LON-151	s__Rickettsia rickettsii
	GCF_002356935	*Rickettsia japonica* str. HH06125	s__Rickettsia rickettsii
	GCF_002356955	*Rickettsia japonica* str. HH06116	s__Rickettsia rickettsii
	GCF_002356975	*Rickettsia japonica* str. HH-18	s__Rickettsia rickettsii
	GCF_002356995	*Rickettsia japonica* str. HH-17	s__Rickettsia rickettsii
	GCF_002357015	*Rickettsia japonica* str. HH-16	s__Rickettsia rickettsii
	GCF_002357035	*Rickettsia japonica* str. HH-13	s__Rickettsia rickettsii
	GCF_002357055	*Rickettsia japonica* str. HH-12	s__Rickettsia rickettsii
	GCF_002357075	*Rickettsia japonica* str. 3416	s__Rickettsia rickettsii
	GCF_002357095	*Rickettsia japonica* str. 2763	s__Rickettsia rickettsii
	GCF_002357115	*Rickettsia japonica* str. Tsuneishi	s__Rickettsia rickettsii
	GCF_002357135	*Rickettsia japonica* str. PO-1	s__Rickettsia rickettsii
	GCF_002357155	*Rickettsia japonica* str. OHH-1	s__Rickettsia rickettsii
	GCF_002357175	*Rickettsia japonica* str. Nakase	s__Rickettsia rickettsii
	GCF_002357195	*Rickettsia japonica* str. MZ08014	s__Rickettsia rickettsii
	GCF_002357215	*Rickettsia japonica* str. HH07167	s__Rickettsia rickettsii
	GCF_002357235	*Rickettsia japonica* str. HH07124	s__Rickettsia rickettsii
	GCF_002357255	*Rickettsia japonica* str. HH06154	s__Rickettsia rickettsii
	GCF_002357275	*Rickettsia japonica* str. HH-1	s__Rickettsia rickettsii
	GCF_002357295	*Rickettsia japonica* str. DT-1	s__Rickettsia rickettsii
	GCF_003454715	*Rickettsia japonica* str. LA42015	s__Rickettsia rickettsii
	GCF_005549115	*Rickettsia parkeri* str. AtlanticRainforest	s__Rickettsia rickettsii
	GCF_007989425	*Rickettsia asiatica* str. Maytaro1284	s__Rickettsia helvetica_A
	GCF_009731485	*Rickettsia heilongjiangensis* str. Sendai-29	s__Rickettsia rickettsii
	GCF_009731505	*Rickettsia heilongjiangensis* str. Sendai-58	s__Rickettsia rickettsii
	GCF_009731525	*Rickettsia heilongjiangensis* str. HCN-13	s__Rickettsia rickettsii
	GCF_009731545	*Rickettsia heilongjiangensis* str. CH8-1	s__Rickettsia rickettsii
	GCF_009857995	*Rickettsia japonica* str. LA162015	s__Rickettsia rickettsii
	GCF_900243065	*Rickettsia fournieri* str. AUS118	s__Rickettsia rickettsii
	GCF_900536895	*Rickettsia typhi*	s__Rickettsia typhi
**Mitochondrion**	KY457529	*Hyalomma truncatum* clone 138/1375_14731_283	
	MT270688	*Hyalomma marginatum* isolate HmarTR4	
	MT270686	*Hyalomma marginatum* isolate HmarTR2	
	MN885800	*Hyalomma marginatum* isolate HmarTR1	
	NC_052828	*Rhipicephalus decoloratus* voucher KBF6	
	MT270687	*Hyalomma marginatum* isolate HmarTR3	

	MW546281	*Hyalomma asiaticum* isolate HasTR1	
	MF101818	UNVERIFIED: *Hyalomma asiaticum* kozlovi	
	MW546280	*Hyalomma aegyptium* isolate HaegypTR1	
	MW366629	*Hyalomma marginatum* isolate HmarTR6	
	MW366631	*Hyalomma marginatum* isolate HmarTR8	
	MW546283	*Hyalomma anatolicum* isolate HanatolTR1	
	MW366630	*Hyalomma marginatum* isolate HmarTR7	
	MW546284	*Hyalomma excavatum* isolate HexTR1	
	KY457528	*Hyalomma rufipes* clone 126/294_14748_295	
	MW366628	*Hyalomma marginatum* isolate HmarTR5	
	MW366632	*Hyalomma marginatum* isolate HmarTR9	
	MF101817	*Hyalomma asiaticum* asiaticum	
	MW366633	*Hyalomma marginatum* isolate HmarTR10	
	NC_053941	*Hyalomma asiaticum* voucher WY042-2	
	MW546282	*Hyalomma scupense* isolate HscuTR1	

Table S2. Microfluidic real-time PCR data for ticks collected from bird species trapped in the Mediterranean basin during the spring migration of 2014 and 2015. The ticks were screened for *Francisella* and spotted fever group *Rickettsia* species using primers targeting the *fopA* and *gltA* genes.

Bird data				Tick data																															
				Ixodes sp.				*Hyalomma marginatum*				*Hyalomma rufipes*				*Hyalomma* sp.				*Heamaphysalis* sp.				*Amblyomma* sp.				ND				Total			
**Bird species**	**Common name**	**Migration distance**	**Tick-infested**	**Nb**	**F**	**R**	**F+R**	**Nb**	**F**	**R**	**F+R**	**Nb**	**F**	**R**	**F+R**	**Nb**	**F**	**R**	**F+R**	**Nb**	**F**	**R**	**F+R**	**Nb**	**F**	**R**	**F+R**	**Nb**	**F**	**R**	**F+R**	**Nb**	**F**	**R**	**F+R**
Charadrius alexandrinus	Kentish plover	Resident/ Short	1					1	1																							1	1		
Erithacus rubecula	European robin	Medium	1					2	2	2	2																					2	2	2	2
Phylloscopus collybita	Chiffchaff	Medium	2	2		1																										2		1	
Turdus philomelos	Song thrush	Medium	1	2																												2			
Acrocephalus schoenobaenus	Sedge warbler	Long	17									53	35	33	26													7	4	6	3	60	39	39	29
Acrocephalus scirpaceus	Eurasian reed warbler	Long	1									1	1																			1	1		
Anthus trivialis	Tree pipit	Long	6									4	4	2	2	3	3							6	5	3	2	6	5	6	5	19	17	11	9
Caprimulgus europaeus	European nightjar	Long	1									1	1	1	1																	1	1	1	1
Falco naumanni	Lesser kestrel	Long	1																	2	2	2	2									2	2	2	2
Ficedula albicollis	Collared flycatcher	Long	6									8	6	6	4													1				9	6	6	4
Ficedula hypoleuca	European Pied flycatcher	Long	21	3	1	1	1					19	19	12	12									2		1		11	7	5	5	35	27	19	18
Hippolais icterina	Icterine warbler	Long	5	2								5	3	3	2																	7	3	3	2
Lanius senator	Woodchat shrike	Long	19					2	2	2	2	45	36	27	22													3	3	2	2	50	41	31	26
Luscinia megarhynchos	Nightingale	Long	7					5	5	2	2	14	9	11	8																	19	14	13	10
Monticola saxatilis	Rufous-tailed rock thrush	Long	1									1	1	1	1																	1	1	1	1
Motacilla flava	Yellow wagtail	Long	1									2		2																		2		2	
Muscicapa striata	Spotted flycatcher	Long	5									4	2	3	2													3	2	1	1	7	4	4	3
Oenanthe oenanthe	Wheatear	Long	5					6	6			21	8	15	6													1		1		28	14	16	6
Oriolus oriolus	Golden oriole	Long	19	6								30	24	26	21													4	2	2	2	40	26	28	23
Phoenicurus phoenicurus	Common redstart	Long	19					2		1		29	21	16	14													1	1	1	1	32	22	18	15
Phylloscopus sibilatrix	Wood warbler	Long	15									21	17	16	14													9	2	3	2	30	19	19	16
Saxicola rubetra	Whinchat	Long	47									123	102	66	60	8	7	2	2									1				132	109	68	62

Streptopelia turtur	Turtle dove	Long	1									3	2	2	2																	3	2	2	2
Sylvia atricapilla	Blackcap	Long	2									4	2	3	1																	4	2	3	1
Sylvia borin	Garden warbler	Long	4									1	1	1	1													4	3	3	3	5	4	4	4
Sylvia communis	Whitethroat	Long	35	2		1		6	2	5	1	56	47	27	25													15	9	12	7	79	58	45	33
Upupa epops	Eurasian hoopoe	Long	1									2	2	2	2																	2	2	2	2
**Total**			**244**	**17**	**1**	**3**	**1**	**24**	**18**	**12**	**7**	**447**	**343**	**275**	**226**	**11**	**10**	**2**	**2**	**2**	**2**	**2**	**2**	**8**	**5**	**4**	**2**	**66**	**38**	**42**	**31**	**575**	**417**	**340**	**271**
%					*5.9*	*17.6*	*5.9*		*75.0*	*50.0*	*29.2*		*76.7*	*61.5*	*50.6*		*90.9*	*18.2*	*18.2*		*100*	*100*	*100*		*62.5*	*50.0*	*25.0*		*57.6*	*63.6*	*47.0*		*72.5*	*59.1*	*47.1*

fopA, Outer membrane protein A gene
gltA, Citrate synthase gene
ND, No data
Nb, Number of ticks
sp., species
F, Number of ticks testing positive for *Francisella* species by microfluidic realtime PCR with primers targeting the *fopA* gene
R, Number of ticks testing positive for spotted fever group *Rickettsia* by microfluidic realtime PCR with primers targeting the *gltA* gene
F+R, Number of ticks testing positive for both *Francisella* and spotted fever group *Rickettsia* species by microfluidic realtime PCR with primers targeting the *fopA* and *gltA* genes

Figures

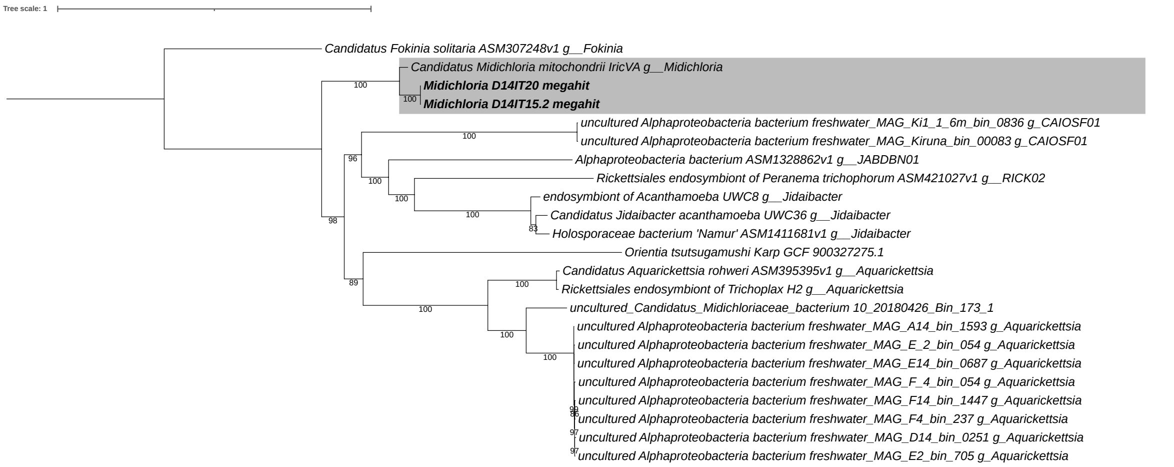

Figure S1. Whole genome maximum likelihood phylogeny. Highlighted area indicates *Midichloria* clade. Study genomes are in bold. *Orientia tsutsugamushi* strain Karp was used to root the tree. Bootstrap values ≥75 are presented at the nodes. The scale bar represents the expected number of substitutions per site.

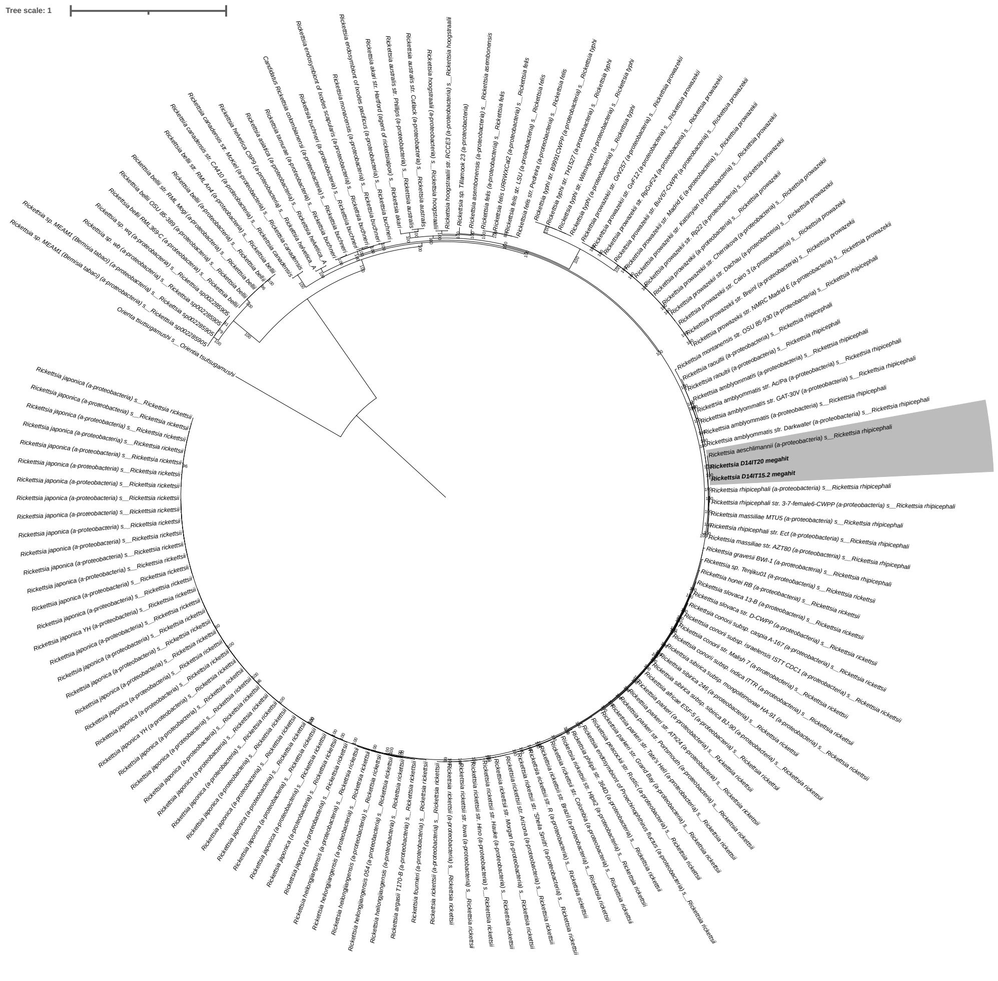

Figure S2. Whole genome maximum likelihood phylogeny of *Rickettsia*. Highlighted area indicates clade for *Rickettsia aeschlimannii*. Study genomes are in bold. *Orientia tsutsugamushi* was used to root the tree. Bootstrap values ≥75 are presented at the nodes. The scale bar represents the expected number of substitutions per site.

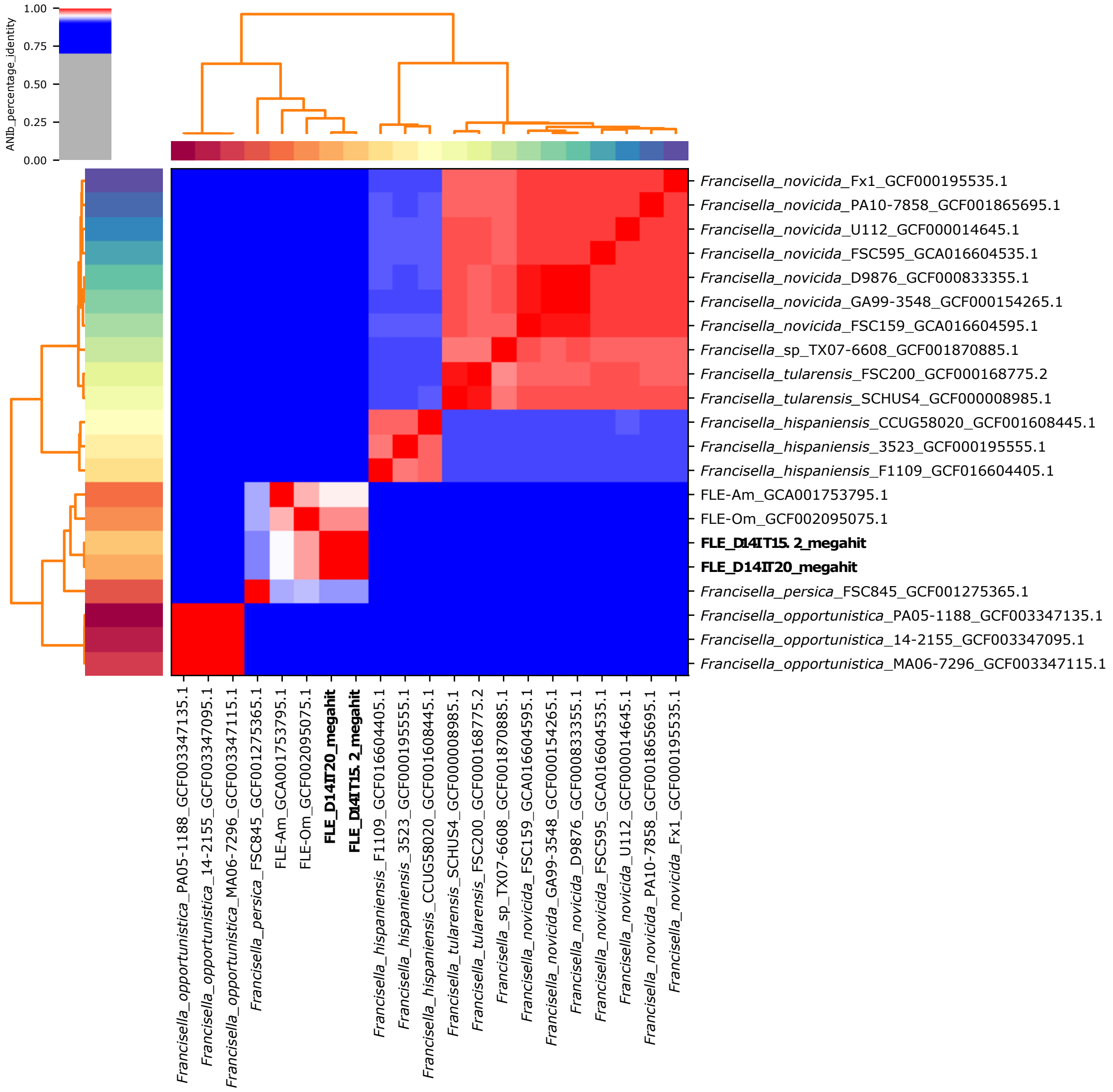

Figure S3. Heatmap of the average nucleotide identity (ANI), demonstrating nucleotide-level genomic similarity between *Francisella* genomes. The pairwise comparison of 19 *Francisella* genomes was computed by BLAST, using the pyANI software. Study genomes are in bold. FLE, *Francisella*-like endosymbiont.

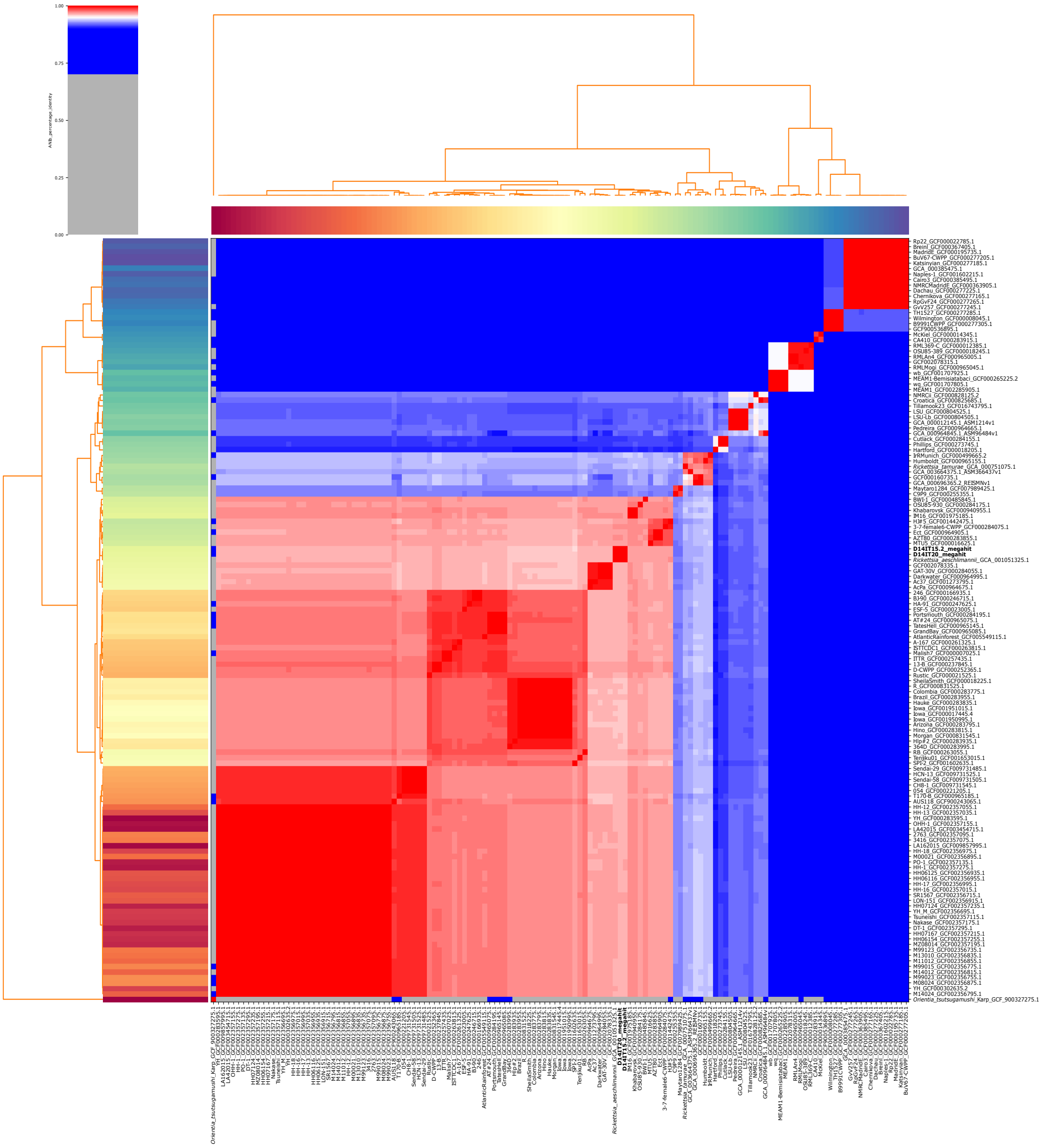

Figure S4. Heatmap of the average nucleotide identity (ANI), demonstrating nucleotide-level genomic similarity between *Rickettsia* genomes. The pairwise comparison of 138 *Rickettsia* genomes was computed by BLAST, using the pyANI software. Study genomes are in bold. For NCBI organism names, see Table S1.

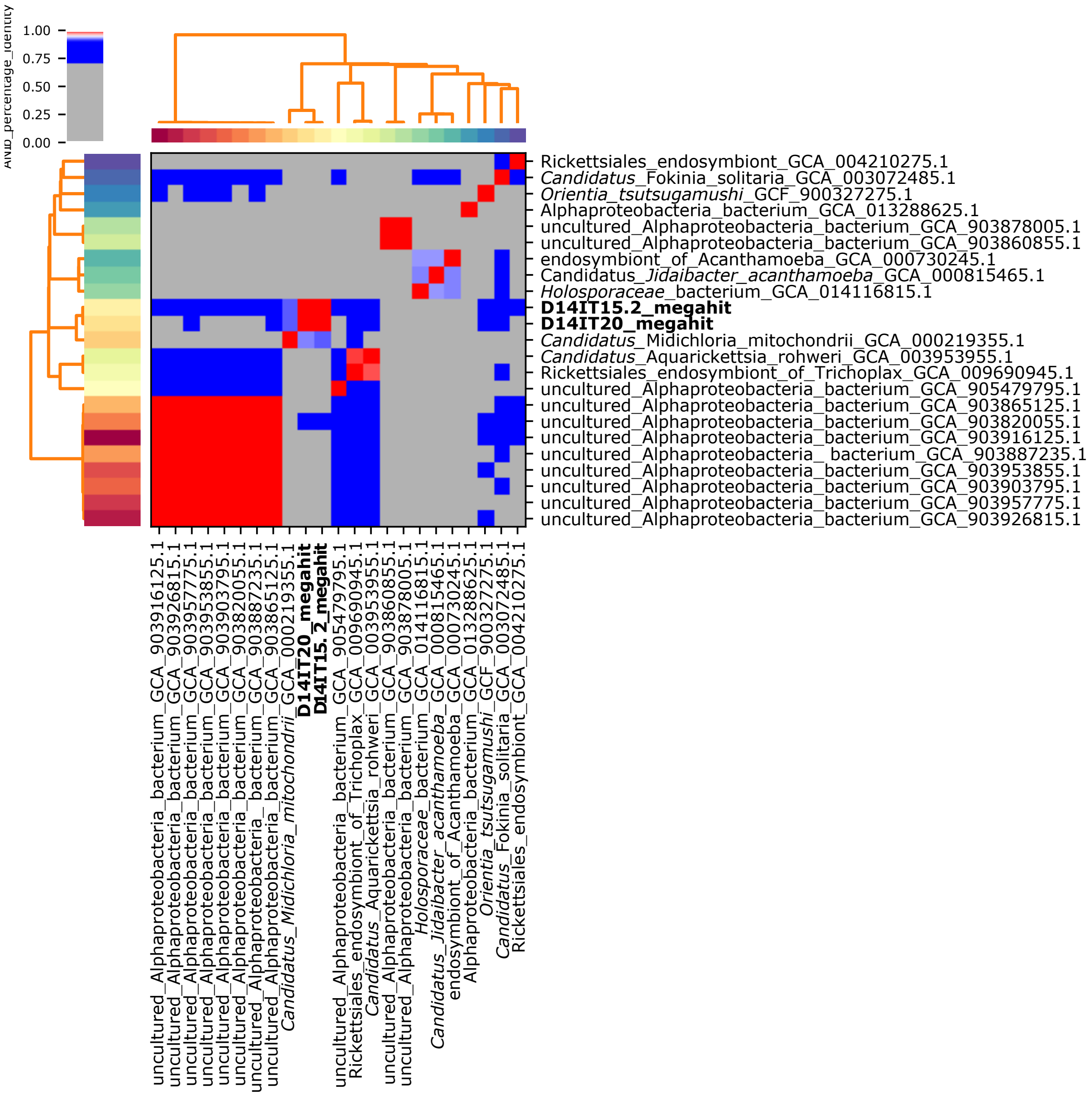

Figure S5. Heatmap of the average nucleotide identity (ANI), demonstrating nucleotide-level genomic similarity between bacterial genomes. The pairwise comparison of 23 genomes was computed by BLAST, using the pyANI software. Study genomes are in bold.

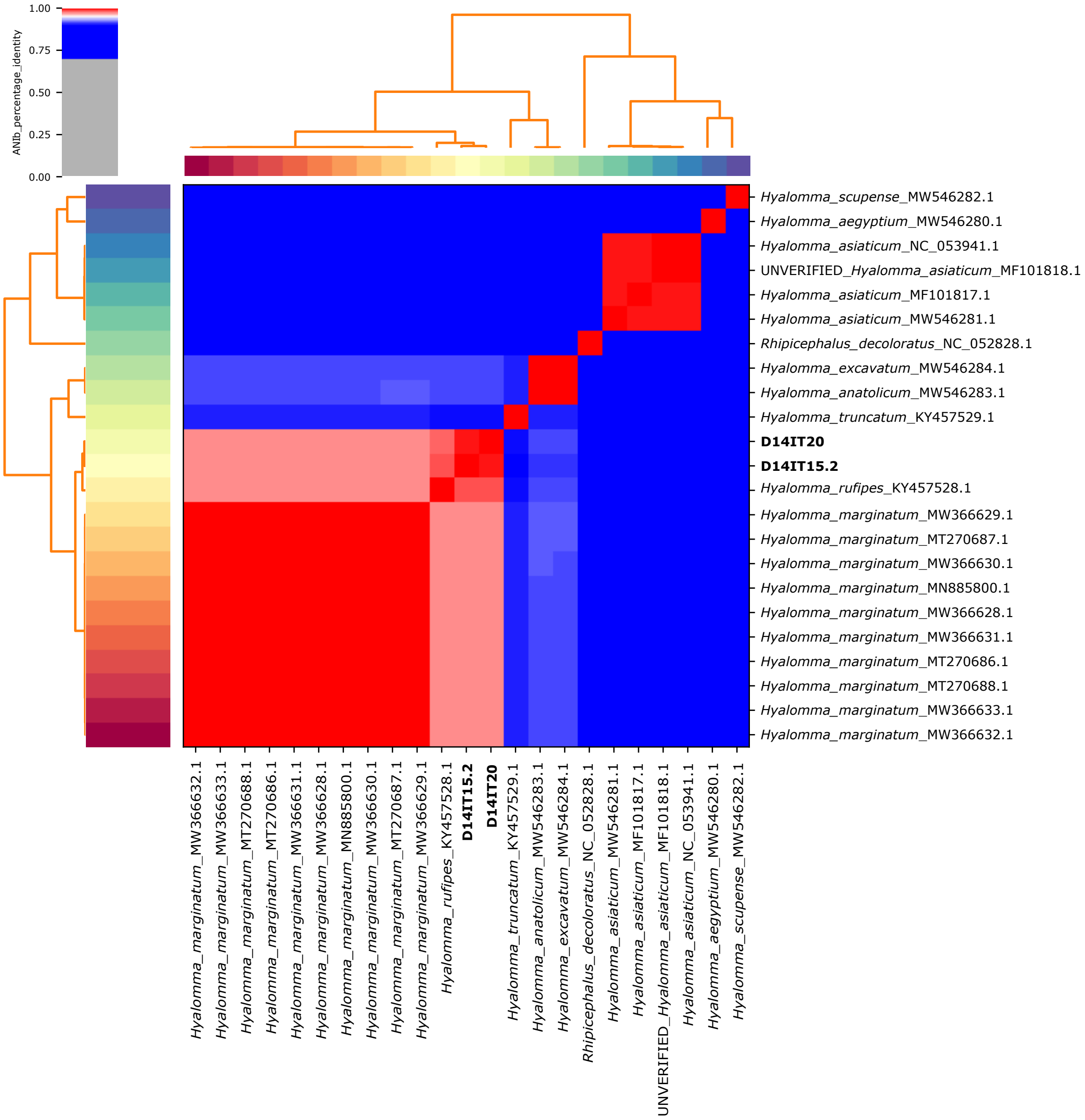

Figure S6. Heatmap of the average nucleotide identity (ANI), demonstrating nucleotide-level genomic similarity between *Hyalomma* mitochondrial genomes. The pairwise comparison of 23 *Hyalomma* mitochondrial genomes was computed by BLAST, using the pyANI software. Study genomes are in bold.
